# Comparison of Physiological Brain Responses Evoked by Visual and Electrical Stimulation

**DOI:** 10.1167/iovs.66.5.1

**Published:** 2025-05-01

**Authors:** Katarzyna Kordecka, Ewa Kublik, Andrzej T. Foik

**Affiliations:** 1 International Centre for Translational Eye Research (ICTER), Institute of Physical Chemistry, PAS, Warsaw, Poland; 2 Institute of Physical Chemistry, PAS, Warsaw, Poland; 3 Nencki Institute of Experimental Biology, Warsaw, Poland

**Keywords:** transcorneal alternating current stimulation (tACS), visual rehabilitation, visual pathway, electrically evoked potentials (EEPs), visually evoked potentials (VEPs), visual dysfunctions, electrophysiology

## Abstract

**Purpose:**

Noninvasive current stimulation (nCS or electrical stimulation) is a rapidly developing technique to support recovery from eye and brain dysfunctions. One of the most commonly used forms of nCS for treating the visual system is transcranial and transcorneal alternating current stimulation. This technique can exert neuromodulatory effects on the brain through eye stimulation. The mechanism of such stimulation is still poorly understood.

**Materials and Methods:**

To understand the pattern of activation evoked by nCS, a series of electrical impulses were delivered directly to the rat eye, alternating with the visual stimulus (VS), and subsequent responses were tested in the Superior colliculus and the primary visual cortex. Additionally, we tested two stimulation electrode placements, eyeball-eyeball, and eyeball-neck.

**Results:**

The results indicate that nCS and VS evoke different activation patterns in the recorded structures. In particular, the electrically evoked potentials are characterized by shorter latency and a different shape than the corresponding visually evoked potentials. The transcorneal alternating current stimulation (tACS) evoked shorter sinks and sources in all recorded structures than the visual stimulation. This suggests emerging of a different pattern of extracellular current flow in response to different stimulations.

**Conclusions:**

We demonstrate that the eye-eye paradigm of electrical stimulation elicited responses more similar to those evoked by VS. Individual transcorneal electrical impulses evoke a consistent pattern of neuronal activation across the visual system. This consistency is particularly promising for the development of neurotherapy aimed at restoring or improving vision, nCS can effectively activate visual circuits despite variations in stimulus delivery and shape.

Noninvasive current stimulation (nCS) has become one of the most popular and quickly developing techniques in treating brain disorders. In recent years, nCS has been applied in numerous studies to improve visual,^1^^–^^11^ motor, somatosensory, or cognitive functions.^12^^–^^14^ One type of nCS is transcorneal alternating current stimulation (tACS), which has become a common tool in treating visual impairment. The tACS is especially used in various retinal degeneration diseases, like retinitis pigmentosa, glaucoma, or optic neuropathy, where it has been proposed to delay the degeneration process and improve visual performance.^1^^,^^2^^,^^4^^–^^9^^,^^11^

The most desired advantage of noninvasive current stimulation is its impact on inducing neuroplasticity in the visual system.^15^^–^^27^ The beneficial effect of nCS is related to improved chorioretinal blood circulation, and increased neurotrophic factors^28^ in the stimulated region, as well as in the central brain areas, through the generation of cortical oscillations.^16^^,^^18^^,^^25^^,^^29^^–^^32^

Despite the increased interest in nCS, little is known about how this technique influences brain activity.^33^^,^^34^ Therefore, it is especially important to find out the mechanisms of electrical stimulation, and its influence on brain cells due to the huge difference between electrical^35^ and visual^36^^–^^38^ rehabilitation training. In this study, we closely examined the shape and properties of the electrically evoked responses to compare them to light-evoked responses in the superior colliculus (SC) and primary visual cortex (VCx). Additionally, we verified the two configurations of electrode placement^34^: (1) stimulating and reference electrodes, both located on the eyeball (E-E); and (2) one electrode on the eyeball and the other on the neck (E-N). As we demonstrate, electrical impulses in the rat’s visual system result in shorter electrophysiological response latencies and distinct shapes compared to visual stimulus. In turn, individual transcorneal electrical impulses elicited a similar pattern of neuronal response in both visual structures.

## Materials and Methods

### Subjects

All experimental procedures were conducted in accordance with the 86/609/EEC Directive and were accepted by the First Warsaw Local Ethical Commission for Animal Experimentation (442/2013). All efforts were undertaken to limit the number of animals used for the study and avoid their stress and suffering. All procedures adhered to the ARVO Statement for the Use of Animals in Ophthalmic and Vision Research. Animals were cared for in accordance with the Animal Welfare Act and the “Guide for the Care and Use of Laboratory Animals.”

The experiments were conducted on 10 (*n* = 10) adult male or female Wistar rats (250–500 g) obtained from The Medical University of Białystok, Poland. Rats were housed in a colony room in the Animal House of the Nencki Institute with food and water available ad libitum and maintained on a 12-hour light/12-hour dark cycle (light on 7:00 AM). Electrophysiological experiments were performed between 9:00 am and 7:00 PM.

### Surgical Procedures

**Figure 1.**
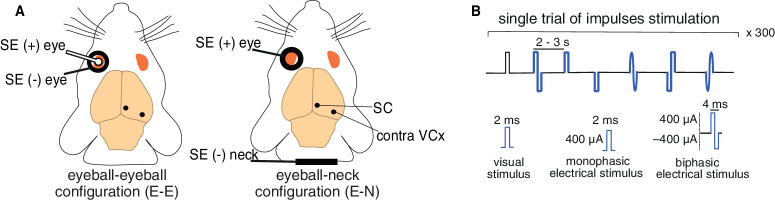
**Electrode placements and experiment design**. (**A**) Two types of stimulating-electrode montages and recording sites. (**B**) Stimulation paradigm and shapes of current impulses used in the experiments.

Rats were anesthetized with urethane (1.5 g/kg; Sigma-Aldrich, Germany; 30% aqueous solution, intraperitoneal [IP]) and placed in a stereotaxic apparatus. The depth of anesthesia was routinely controlled by checking for the presence of a withdrawal reflex and by monitoring the electrocorticogram (ECoG). When high-frequency, low-amplitude activity dominated the ECoG, an additional dose of urethane (0.15 g/kg) was administered. Body temperature was maintained at 37°C to 38°C using an electrical heating blanket with automatic control (Harvard Apparatus), and liquid requirements were fulfilled by subcutaneous 2-mL injections of 0.9% NaCl (Polfa Warszawa S.A., Poland) every 2 to 3 hours. Corneas were lubricated with Lacrimal (Polfa Warszawa S.A., Poland), as required to prevent drying. The skin on the head was covered with iodine, and local anesthetic (lidocaine hydrochloric 2%, 0.3 mL; Polfa Warszawa S.A., Poland) was injected subcutaneously along the incision line. The skull was exposed and trephined (approximately 3 mm in diameter) in two areas (see Fig. 1) overlaying the binocular VCx, contralateral to the visually or electrically stimulated eye = 6.0 to 7.5 mm posterior to the bregma, 4.0 mm lateral from the midline; and the contralateral SC = 7.0 mm posterior to the bregma, 1.5 mm lateral from the midline. Stereotaxic coordinates were based on the rat-brain atlas of Paxinos and Watson.^39^

### Local Field Potential

Continuous spontaneous local field potential (LFP) and visually evoked potentials (VEP) or electrically evoked potentials (EEP) were recorded in all animals from two structures of the visual system simultaneously: contralateral SC and primary VCx.^34^ For the recordings, we used monopolar, custom-made linear tungsten microwire electrodes (California Fine Wire, USA, 25 µm tungsten in HML insulation) or silicon-probe electrode arrays (NeuroNexus Technologies, USA) with an Ag/AgCl ground-reference wire positioned in the neck muscles. SC recording probes comprised 7 wires with a vertical recording site separation of approximately 200 µm. This arrangement of recording sites increased the chances of successfully recording from the desired structure. Cortical recordings were made using 16-channel silicon probes with a recording-site distance of 150 µm. Electrode tips were lowered to 2.1 mm and 4 mm from the cortical surface to record from the VCx and SC, respectively. Locations of the electrodes were confirmed histologically postmortem, as we described previously.^34^ The signals were bandpass filtered between 0.3 and 5 kilohertz (kHz) and amplified (500×) using 16-channel differential AC amplifiers (A-M Systems, USA). Recorded signals were digitized (10 kHz sampling rate) and fed to a personal computer for online display, analysis, and data storage via a CED Power 1401 system (Cambridge Electronic Design, UK) and a multichannel data acquisition system (USB-ME256-Systems, Multichannel System, Germany), along with Spike2 software (Cambridge Electronic Design, UK). Visual and electrical stimulation marks were recorded along with the electrophysiological signals in the same data file.

### Visual Stimulation

VEPs recorded from the contralateral SC and VCx were obtained in response to flashing white-light-emitting diodes (LEDs) placed 10 cm in front of the left eye. The stimulus (7600 cd/m^2^ luminance, 2-ms duration) was repeated 300 times, intermingled with transcorneal electrical stimulation (see Fig. 1).

### Transcorneal Alternating Current Stimulation and Experimental Design

The electrical stimulation procedure was performed similarly to our previously published research.^34^ Two different stimulating-electrode configurations were tested: eyeball-eyeball and eyeball-neck (see Fig. 1A). The eye-eye configuration consisted of two electrodes, an Ag/AgCl wire (0.2 mm thick) ring (5 mm inner diameter) placed on the cornea, and an Ag/AgCl ball (1 mm diameter) placed inside the ring (see Fig. 1A, left panel). The eyeball-neck configuration consisted of a corneal bulb electrode and an Ag/AgCl wire placed in the neck muscles (see Fig. 1A, right panel).

The tACS consisted of rectangular and sinusoidal biphasic current pulses, and monophasic with negative and positive pulses applied randomly. Biphasic impulses (2 ms per phase) were 800-µA amplitudes in total (±400 µA each phase), whereas monophasic impulses were 400 µA. Pulse parameters that produced a clear EEP were optimized during the first experiment and then used for all subsequent experiments. Single pulses were delivered by a CED Power 1401 system, using Spike2 software (Cambridge Electronic Design, UK) and a linear stimulus isolator unit (World Precision Instruments, Sarasota, FL, USA). We have used six variants of single electrical impulses, which differed in the shape of the impulse: rectangular biphasic impulse starting from positive or negative component (RPB or RNB); rectangular positive and negative monophasic impulses (RPM and RNM); and single sinusoidal impulse starting from the positive or negative phase (SPB or SNB; see Fig. 1B)*.* The experimental paradigm was the same for 10 rats. One stimulation trial consisted of one series of impulses (1 visual and 6 electrical) separated by random interstimulus time intervals (2–3 seconds), and it was repeated 300 times for the 2 stimulation electrode montages, eyeball-eyeball and eyeball-neck (see Fig. 1B).

### Histology

Standard histological techniques were used to verify the placement of the electrodes. Electrodes coated with DiI (1,1′-dioctadecyl-3,3,3′,3′ tetramethyl-indocarbocyanine perchlorate; Sigma-Aldrich, Germany) were used in some cases to facilitate electrode tract reconstruction. Rats were injected with an overdose of Nembutal (150 mg/kg) at the end of the experiment and perfused through the heart with 4% paraformaldehyde in phosphate-buffered saline. The brains were removed, stored in paraformaldehyde and 30% sucrose for cryoprotection, then cut into 50 µm slices, and stained with cresyl violet.

### Data Analysis and Statistics

All offline data analysis was performed using MatLab (MathWorks) custom-written scripts. All data sets obtained from each animal were normalized by a commonly used z-score standardization method; that is, the mean signal value of all recordings made for a single rat was subtracted from each data point, which was then divided by the signal standard deviation calculated for all recordings from a single animal. Interference and noise in the data were removed with the Chronux toolbox,^40^ and the signal was then filtered with a low pass filter (120 hertz [Hz] cutoff frequency) and down-sampled to 1 kHz. Differences between the responses were assessed by comparing the peak-to-peak amplitudes of the evoked potentials. Results are given as the mean ± SEM. The Mann-Whitney *U* test was used for pair-wise comparisons to verify any statistically significant differences in the amplitudes of the evoked potentials. Differences were considered significant at *P* ≤ 0.05 for two-tailed tests.

**Figure 2.**
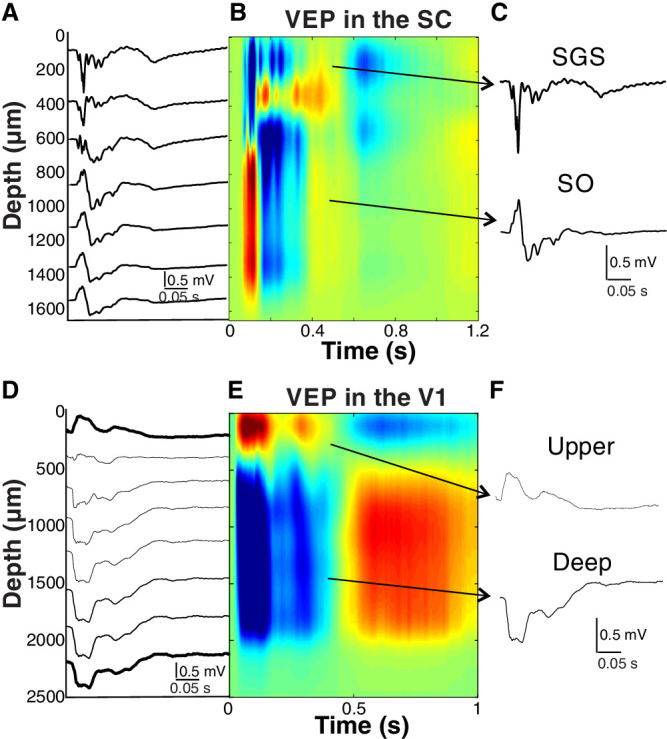
**Collicular and cortical visually evoked profiles**. The analysis profiles of the SC and the VCx profiles were simplified by picking evoked potentials from superficial and deep layers of both structures. (**A**) Evoked-potential profile recorded in the SC. (**B**) CSD profile computed from potentials shown in **A**. (**C**) Example of the responses chosen from the SGS and SO layers. (**D**) Evoked-potential profile recorded in the VCx. (**E**) The CSD profile was computed from the potentials shown in **D**. (**F**) Two typical responses were chosen from upper and deep layers in the VCx.

The layered structure of the SC and VCx can be represented as the current source density (CSD) profile for a more detailed representation. The kernel CSD analyses were performed according to the method introduced by Potworowski et al.^41^ The characteristic profiles allowed us to distinguish different layers due to intermingled current sinks and sources compositions (Figs. 2B, 2E), also visible as “switching dipoles.” For further analysis, we selected channels specific to each layer, as shown in Figures 2C and 2F. Based on the dipole pattern, we have peaked one signal from the superficial layers of the SC and V1 and one from the deep layers.

The average Pearson correlation was calculated to assess the similarity between VEP and EEPs. The similarity of the responses was quantified as the Pearson correlation between each pair of tested impulses in the entire response window (1.1 seconds). The amplitude is defined as the difference between the maximum value of the positive component in the response and the minimum value of the negative component in the response. The response latency is the maximum signal value taken from the absolute value of the entire response window.

## Results

Electrophysiological LFP recordings were done in 10 albino rats (Wistar). To cover multiple layers of each structure, signals were collected by multielectrode arrays placed simultaneously in two structures of the visual system: the Stratum Griseum Superficiale (SGS) and Stratum Opticum (SO) layers (see Figs. 2A, 2B, 2C) of the SC, upper and deep layers of the primary VCx (see Figs. 2D, 2E, 2F). Each structure can be characterized by different response shapes or CSD patterns to visual or current stimulation.

**Figure 3.**
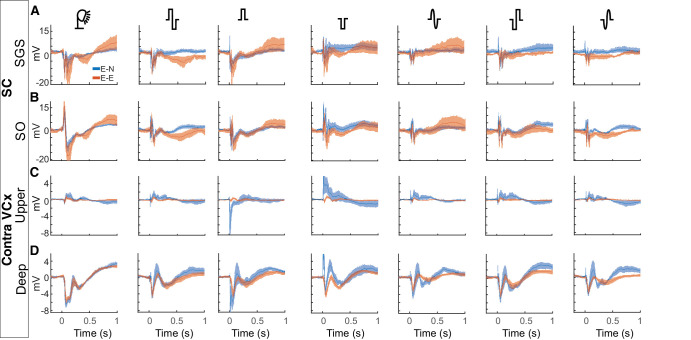
**Average visually and electrically evoked potentials with various electrode arrangements**. (**A**) VEP and EEPs recorded in the SGS of the SC. (**B**) SO of the SC. (**C**) Upper layers of the VCx. (**D**) Deep layers of the VCx. Shadings indicate SEM.

In Figure 3, we present the average evoked potentials (*n* = 10) recorded from the SGS and SO layers of the SC (see Figs. 3A, 3B) and the upper and lower layers of the visual cortex (see Figs. 3C, 3D). The potentials are shown in the columns corresponding to those evoked by light (left-most column), or by various electrical impulses: rectangular biphasic impulses, positive and negative monophasic impulses, and sinusoidal biphasic (positive and negative) impulses. The data for the two electrode montages are presented in rows: eye–eye (E-E), eye–neck (E-N), and all recorded structures.

### The Different Electrical Stimuli Evoke Similar Response Amplitudes

**Figure 4.**
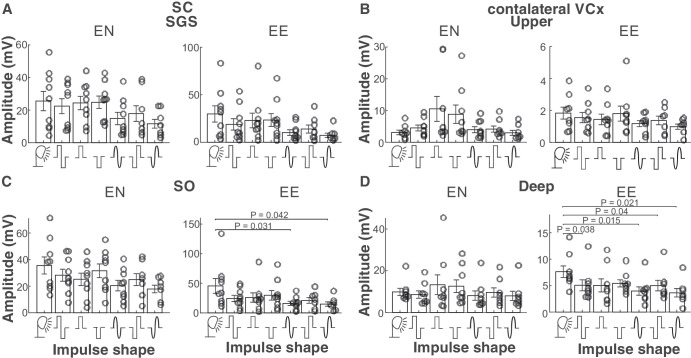
**Population average amplitudes for V1 and SC for both layers**. (**A**, **C**) Average VEP and EEP potential amplitudes in the SGS and SO of the superior colliculus. (**B**, **D**) Average VEP and EEP potential amplitudes in the supra- and infragranular layers of the contralateral visual cortex.

Potentials evoked by electrical stimulation (Fig. 4) in the E-N configuration were generally comparable in amplitude with the VEPs. We did not find a significant difference in potential amplitudes. The E-E configuration elicited more diverse EEP responses. We found a significant difference between EEP and VEP for both sinusoidal impulses in the SC (*P* = 0.031 and *P* = 0.042) and in the VCx (*P* = 0.015 and *P* = 0.021). Moreover, we also found smaller response amplitudes evoked by both rectangular impulses in the VCx.

### Assessment of the Similarity of Electrically Evoked and Visually Evoked Potentials

**Figure 5.**
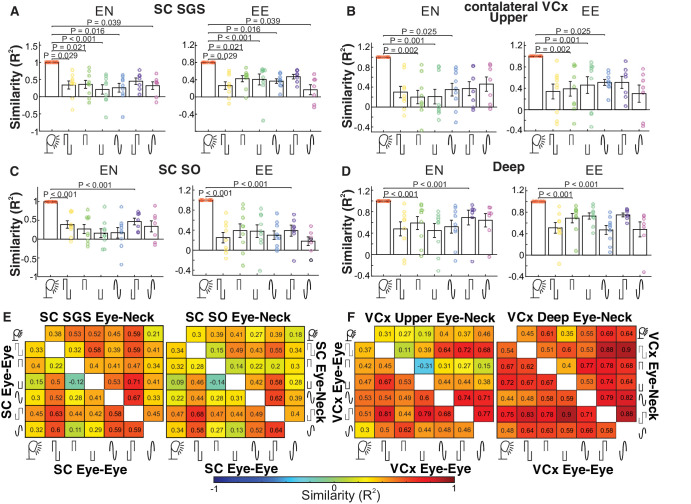
**Assessment of the similarity between electrically and visually evoked potentials**. The similarity was measured as a waveform correlation coefficient for the two stimulating paradigms, eye-eye and eye-neck, for all recorded structures and layers. The average similarity between VEPs and EEPs of the SC for the SGS layer (**A**), and the SO layer (**C**). The average similarity of the VEPs and EEPs are shown for the upper layers (**B**) and the deep layers (**D**) of the contralateral visual cortex. (**E**) Heatmap representations of relative similarities of the VEPs and EEPs of the SGS and SO layers in the SC. (**F**) Similarity heatmaps represent the analysis of the evoked potentials from the visual cortex.

As shown in all structures, EEPs significantly differed in shape from VEPs. To quantify such differences, we calculated an average Pearson correlation to measure the similarity between VEP and EEP waveforms (Fig. 5). In the SC, the electrical impulses delivered by both montages evoked significantly different responses than the flash of light. The electrically evoked responses of the E-E montage were least similar to the VEP, and only the E-N montage values exceeded a similarity of 0.5. In other words, the electrical stimulation evoked rather different responses than the visual stimulus in the SC. In contrast, we observed different scenarios in the V1, where, in general, responses were more similar to visual stimulation, especially in the deep layers. Generally, the correlation in the deep layer of the VCx was higher than in the upper layer, or in the SC. The detailed analysis of each stimulus similarity is shown in the heatmaps in Figures 5E and 5F.

### Response Latency for Different Electrical Stimuli

**Figure 6.**
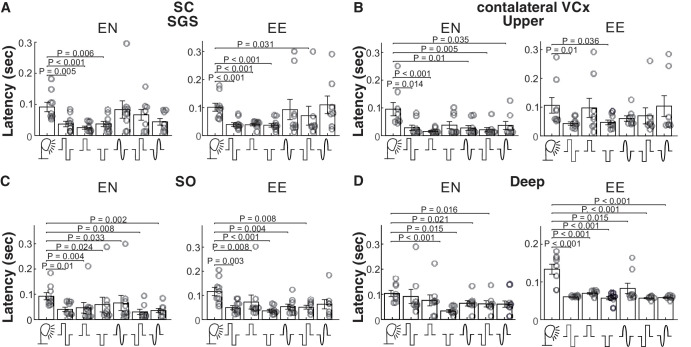
**Population data showing average maximum peak latency for visual and electrical evoked potentials for all recorded structures and layers**. (**A**) Average latencies for VEP and EEP in the SGS of the SC for the E-N electrode montage (*left columns*), and the E-E montage (*right columns*). (**B**) Average latencies for VEP and EEP in the upper layer of the contralateral VCx are shown, analogous to **A**. (**C**) Average latencies for VEP and EEP in the SO of the SC. (**D**) Average latencies for VEP and EEP in the deep layer of the contralateral VCx.

We have measured the response latency of the maximum-peak amplitude evoked by specific stimulation. In all cases of the electrical stimulation, the peak response latency was shorter for electrical than for visual stimulation. Positive and negative monophasic stimulation created the shortest latency (Fig. 6); although those impulses also created the most robust amplitude of the response, they simultaneously were the least similar to the response created by visual stimulation.

### Effect of tACS on Current Source Density 

**Figure 7.**
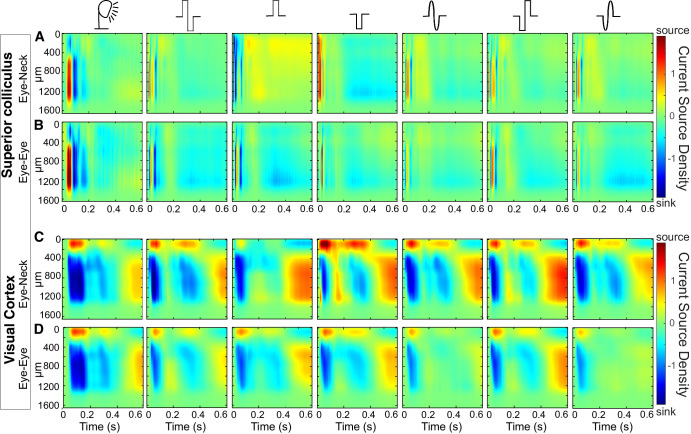
**Examples of CSD profiles from one rat in response to** **seven** **different stimuli**. (**A**) CSD profile recorded in the SC with the E-N paradigm. (**B**) CSD profile recorded in the SC with the E-E paradigm. (**C**) CSD profile recorded in the contralateral VCx with the E-N paradigm. (**D**) CSD profile recorded in the contralateral VCx with the E-E paradigm.

Each impulse shape creates various neuronal activation patterns. To determine how neuronal activation was represented in the distribution of sinks and sources in the layered structures, we calculated CSD profiles from the SC and the contralateral VCx, as shown in Figure 7. This analysis revealed that not only was the latency shorter (see Fig. 6), but also the dynamics of the response to electrical stimulation was faster, resulting in a shorter duration of sinks and sources than in the CSD plotted for the VEPs. This effect is particularly evident in the cortex, where the double peak of the negative wave of light-evoked potential is lost in the EEP, where the two peaks seem to merge in time. Another noticeable effect occurs after monophasic stimulation in the E-N configuration when the negative or positive charge of the impulse causes a reverse response in the upper layers of the VCx and along the entire SC. Moreover, switching the polarization of biphasic pulses does not change the response pattern.

## Discussion

The current study investigated the effects of different types of electrical impulses on the responses of the rat’s visual system. The characteristics of the EEPs were compared with those of a flashing light. Studies have shown a considerable difference in the properties of the reactions of the visual system to light and electrical stimuli. Primarily, potentials evoked by electrical impulses exhibited notable differences in shape compared to VEPs (see Figs. 3, 5, 7). However, the amplitudes of the visual and electrical potentials are similar (see Fig. 4). Additionally, the responses to an electrical impulse are much faster than those to a visual impulse (see Figs. 6, 7). This result was expected because electrical stimulation directly elicits responses from the inner retinal neurons, bypassing the phototransduction process.^42^ Considering the two configurations of stimulating-electrode positions, we find that the E-E paradigm of electrical stimulation elicited responses more similar to those evoked by visual stimulation. Such effect differences were also reported in tACS.^33^

Numerous investigations have delved into the application of tACS in visual disorders caused by eye or brain pathology, as evidenced by the works of Morimoto et al.,^10^ Fedorov et al.,^2^ Sergeeva et al.,^8^ Fu et al.,^1^ and Jassim et al.,^43^ and many more.^44^^,^^45^ These studies show how tACS promotes both morphological and functional changes of surviving retinal cells in various eye diseases. However, there has been comparatively little research into the effects of tACS on the electrophysiology of the visual system to single and very short stimulation. Despite the use of low current intensities (400–800 µA), electrical stimulation of the retinal ganglion cells results in the transmission of the signal to the subcortical structures and then to the visual cortex. Therefore, tACS affects the retina directly and can also modulate neuronal and brain plasticity. Even with increased interest in electrical stimulation, we still need to gather more knowledge about fundamental mechanisms related to activating neuronal circuits, especially because many different paradigms of such stimulation are used in research and human treatments.^46^ The efficacy of tACS can be optimized by adjusting the stimulation parameters, such as current amplitude, stimulation frequency, or electrode placement.^25^^,^^33^^,^^47^^–^^49^

In this study, we more closely examined the shape and properties of the electrically evoked responses to compare them to the light-evoked responses. We considered two electrode configurations and found that the location of the stimulation reference is important and has an impact on the activation of the visual system. Although many studies have tested the efficacy of electrical stimulation, to our knowledge, no studies have systematically compared EEPs with VEPs. We demonstrated that if a single stimulation electrode is placed on the cornea, the EEP comes from the retina, and the current most likely flows through the entire visual system^42^; searching the literature, we found very few reports providing insight on the information processing evoked by various single electrical impulses. Based on data presented in our current article and the literature,^25^^,^^33^ electrical stimulation can evoke different responses using different electrode montages (transcranial versus transcorneal or transorbital).^9^^,^^48^^,^^50^^–^^52^ Ideally, to positively affect vision, we expect to stimulate the visual system electrically in a manner most similar to the natural way of light stimulation, but with enhanced healing properties.^9^^,^^46^ While designing the electrical stimulation paradigms, we must consider the effect of electrode placement, the spatial range of the current, and the strength of the impulse. Such technical considerations, however, are not the only concern. It is also critical to consider the anatomy of the visual system and its responsiveness to various stimuli. In the case of visual stimulation, the light is converted into current in the phototransduction cascade, and processing of the initial stimulus appears one layer after another. In the case of electrical stimulation, we simultaneously stimulate all cells in the neural retina, which we showed as a stronger synchronization (faster processing) in structures upstream in the visual system (SC and VCx). Electrical stimulation omits the sequential processing happening in the retina under native conditions,^53^ even if the stimulation field is limited to the eyeball in the eye-eye montage. In the eye–neck montage, there is a more substantial impact on the visual pathways, which may have different functionality, especially if the disease effects have an impact beyond the retina. Knowing how the current flowing from the eye almost simultaneously stimulates other structures in the visual system may be crucial knowledge for the success of any therapy based on electrical stimulation. Thus, as a take-home message from our findings, any transorbital electrical stimulation paradigm should consider the real impact of the electrical field and the affected brain areas in order to deliver therapy in a precise manner.
