# Erratum in: Comparison of Physiological Brain Responses Evoked by Visual and Electrical Stimulation

**DOI:** 10.1167/iovs.66.6.46

**Published:** 2025-06-13

##  

***Erratum in:*** “Comparison of Physiological Brain Responses Evoked by Visual and Electrical Stimulation” by Katarzyna Kordecka, Ewa Kublik, and Andrzej T. Foik (*Invest Ophthalmol Vis Sci*. 2025;66(5):1), https://doi.org/10.1167/iovs.66.5.1.

The article was first published with the Creative Commons Attribution-NonCommercial-NoDerivatives license. The license should have been the Creative Commons Attribution license. The article has been corrected online.

